# Assessing infant sleep quality and arousals: comparison of wearable data with parent perspectives

**DOI:** 10.1038/s41390-025-04649-y

**Published:** 2025-12-10

**Authors:** Sofie de Sena, Manu Airaksinen, Saeed Montazeri, Elisa Taylor, E. Juulia Paavonen, Sampsa Vanhatalo

**Affiliations:** 1 BABA Center, Paediatric Research Center, Department of Clinical Neurophysiology, Children’s Hospital, Helsinki University Hospital and University of Helsinki, Helsinki, Finland; 2 Department of Physiology, University of Helsinki, Helsinki, Finland; 3 Child Psychiatry, Paediatric Research Center, University of Helsinki and Helsinki University Hospital, Helsinki, Finland; 4 Public Health and Welfare, Finnish Institute for Health and Welfare, Helsinki, Finland

## Abstract

**Background:**

Infant sleep is vital for development, yet current assessments rely on subjective parental reports, underscoring the need for more reliable, objective methods.

**Methods:**

A total of 104 infants (424 nights) were measured at home using a novel NAPping PAnts (NAPPA) wearable, with arousals detected across a wide range of duration criteria. Parental sleep diaries were collected to capture perceived sleep quality (pSQ), reported arousals, and whether the infant slept in a shared or separate room. Diary data were used to study correlations with NAPPA-measured infant arousals, and regression models assessed the relationship between pSQ and reported versus measured arousals.

**Results:**

The pSQ correlated moderately with both the reported (ρ = −0.47, *p* < 0.001) and NAPPA-measured (ρ = −0.40, *p* < 0.001) arousals. The number of reported arousals was most comparable with the NAPPA-measured arousals in the 3–5-min duration range. However, parents significantly underestimated shorter arousals and overestimated longer ones, especially in a shared room with parents.

**Conclusion:**

Direct infant measurements with the NAPPA wearable revealed a substantial difference between directly measured and parentally reported arousals. The findings suggest that infant sleep studies should incorporate direct and objective infant measurements, now made feasible and scalable through novel at-home wearable methods.

**Impact:**

- Infant sleep assessments typically rely on parent’s subjective reports rather than objective measurements of the infant’s sleep.
- This study addresses a critical gap by comparing parental reports of infant sleep with high-resolution, objective measurements from the novel NAPPA (NAPping PAnts) wearable, demonstrating its feasibility for at-home use.
- Specifically, we show that parentally reported nighttime events, such as sleep quality or infant arousals, only partially reflect the infant’s actual sleeping behaviour.
- Our findings highlight the importance of measuring infant sleep objectively, and the novel methodology opens new research avenues that may guide sleep health interventions.

## Introduction

Sleep during infancy is important for neurodevelopment and serves as an indicator of a child’s overall well-being, including their interactions with family and environment. Parental expectations and issues felt around infant sleep may vary widely between individuals, as well as across cultures and life situations.^1,2^ In the general health care practice, a very high proportion of parents report poor sleep quality,^3,4^ which may lead to major stress to the whole family. Poor parent-reported sleep quality in infants is often associated with fragmented sleep, which is implicitly assumed to reflect interrupted nighttime sleep.^5,6^ However, parental reports are prone to bias and may either overestimate or underestimate the frequency of infants’ arousals and awakenings.^7,8^ Studies comparing co-sleeping and solitary sleeping infants suggest that parents’ perception of infant sleep quality is influenced not only by the higher frequency of arousals typically associated with co-sleeping, but also by cultural beliefs about infant sleep and caregiving, as well as parental mental health and the mother’s perception of mother-infant relationship.^9,10^ Together, these findings suggest that parental perceived sleep quality (pSQ) is not directly determined by nighttime arousals, but is likely shaped by a range of other factors, such as family sleeping arrangements, parental expectations and well-being, and their understanding of the infant’s sleep behaviour.^6,10–13^ Therefore, it is evident that a broader use of objective and quantitative methods alongside parental reports is needed to better assess sleep from the infant’s perspective.

When there is no suspicion of physical illness, such as sleep-disordered breathing, an infant’s sleep is assessed indirectly through parental interviews or sleep diaries.^14^ These methods are inherently subjective and reflect only the parent’s perspective. It is well known that even numerical assessments (such as sleep duration, or the timing and number of arousals) are often inaccurate,^15,16^ and their accuracy likely declines when the informant is tired or stressed.^17^ In this context, measuring infant sleep directly could significantly improve the accuracy of its evaluation. The prior studies performed to this end typically rely on measuring infants’ movements with a wrist- or ankle-worn actigraphy, which records sedentary behaviour at a relatively coarse time scale (from minutes to hours). While these studies have highlighted the discrepancies between parental and infant-derived information,^12,16,18^ they both have limitations compromising their reliability as the ultimate benchmark or source of information. Consequently, there is a growing need for more refined and objective approaches to assess infant sleep more reliably.

In this study, we describe the development and characterisation of a novel infant wearable, NAPPA (NAPping PAnts),^19,20^ designed to objectively monitor infant sleep through at-home measurements. In particular, we evaluated how NAPPA measurements could be used to quantify arousals of varying lengths and benchmarked our findings against sleep diaries, which are routinely used to collect parents’ perceived infant sleep quality (pSQ) and their perceptions of infant nighttime arousals.

## Methods

Infants’ nighttime sleep was assessed over three consecutive nights using both parental sleep diaries (subjective assessment) and the NAPPA (NAPping PAnts) wearable. The study included three levels of analyses: First, we used sleep diaries from the parents to assess the relationship between perceived sleep quality (pSQ), reported arousals (RAs), and the sleep setting (shared vs. separate). Second, we systematically assessed infants’ movements of different durations and postural changes from NAPPA measurements to determine how different types of NAPPA arousals (NAs) correspond to the arousals reported by the parents. Third, we studied how the NAs correlate with parents’ assessments of sleep quality (pSQ) and whether this correlation depends on the sleeping setting. Together, these analyses aim to identify objective metrics of infants’ sleeping behaviour that correlate with pSQ.

### Participants

A total of 108 infants contributed sleep measurements to the present study. The infants were initially recruited for a larger neurodevelopmental study (named “PILKE”) at the New Children’s Hospital, Helsinki University Hospital, Finland. Sleep measurements were offered to the infants if they met the eligibility criteria: they were free of any sleep concerns reported by the parents or other health issues that could potentially affect their sleep at the time of recruitment or during the study. Each eligible infant was asked to contribute sleep measurements for one to three nights. For logistical reasons related to other visits to the research unit, sleep measurements were offered during the seven- and twelve-month visits. All sleep assessments were analysed independently of the other neurodevelopmental data, and no longitudinal assessments within sleep domains or across developmental domains were planned. The final dataset in this paper included infants born prematurely (less than 37 weeks gestational age), term-born infants with a clinical suspicion or diagnosis of mild perinatal asphyxia, and healthy term-born infants with no clinical incidents (measurement ages mean 7.4 ± 0.46 and 12.4 ± 0.45, respectively). The larger neurodevelopmental study was reviewed and approved by the relevant Ethics Committee of the New Children’s Hospital, Helsinki University Central Hospital.

### Sleep measurements

#### NAPPA wearable measurements

**Fig. 1:**
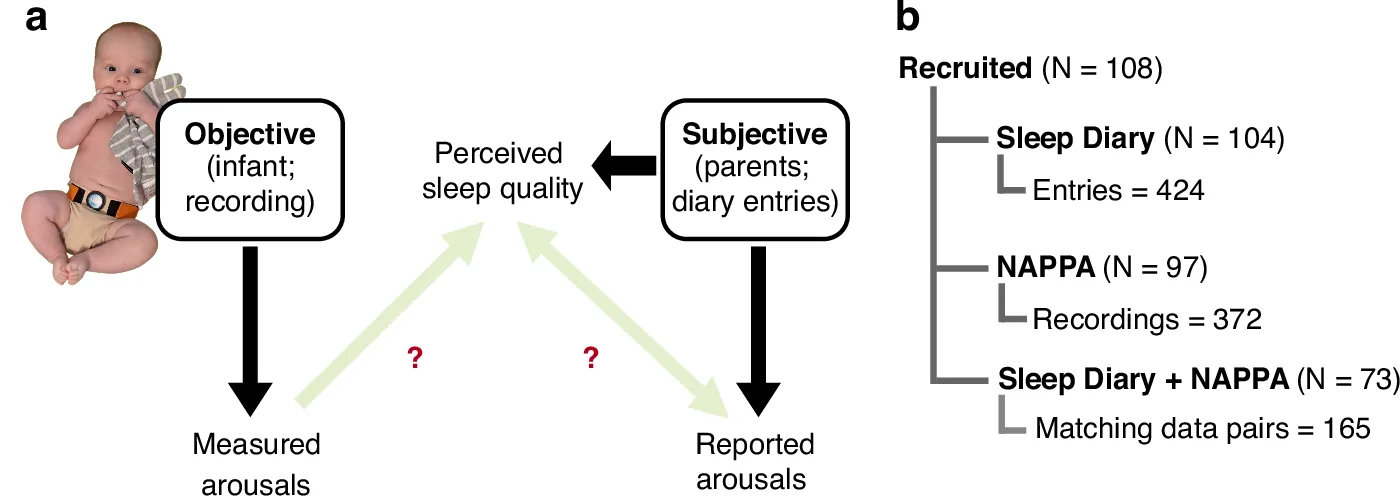
Overview of datasets and rationale for comparing subjective and objective sleep data in infants. **a** Schematic illustration of the rationale for studying infants’ sleep using two complementary sources of information: subjective parental assessment (sleep diary) and objective infant-based measurement (NAPPA). Black arrows indicate data collected in this study; light green arrows represent achievable questions addressed in this study. **b** Flowchart summarising the datasets available for different analyses.

Infants’ empirical sleeping was measured with the novel NAPPA (NAPping Pants) wearable, which enables easy and minimally disruptive nighttime measurements at home. NAPPA is essentially a pull-up diaper cover with a detachable and programmable movement sensor located at the front waistline (see Fig. 1a). When the pants are tightened firmly on the infant, the sensor accurately measures the infant’s body position, movements (akin to actigraphy), and several respiration-related parameters, which were used individually in the present work, while they can also support the assessment of sleep architecture. The body position and activity measures are computed and stored in the sensor’s memory every 5 s using accelerometer signals, while the respiration parameters are calculated and stored every 30 s using gyroscope signals. The measurements are initially stored on the sensor and downloaded to a mobile device using a custom-tailored Nappalogger app. In the present study, we recorded data over three consecutive nights, using 12-h windows (typically from 7 PM to 7 AM) each night.

#### Sleep diary

To assess infants’ sleep from the parents’ perspective during each NAPPA-recorded night, parents were asked to complete a sleep diary similar to the form used in clinical workups at our paediatric sleep clinic (see Supplementary Fig. S1). The questions included hourly sleep, logs of arousals and other events, bed and wake-up times, as well as the parents’ rating of perceived sleep quality (pSQ; score 0–10). The sleep setting (shared vs. separate room) was asked about retrospectively via email 6–33 months later (mean = 20, median = 22).

#### Measurement protocol

During families’ visit to the research unit for other reasons, families were provided with the NAPPA pants. They were instructed to dress their infants in the NAPPA pants over their diapers at bedtime and to remove them upon waking in the morning. Sleep diaries were filled out using pen and paper. A courier service collected the NAPPA pants and diaries and delivered them to the research unit for data analysis. In total, 97 infants used the NAPPA pants at some point during the study (*N* = 72 at 7 months; *N* = 69 at 12 months; *N* = 44 at both time points). Sleep diaries were completed by 77 families at 7 months and by 74 families at 12 months, with 42 families filling out diaries at both time points. For more detailed breakdown of infants and nights recorded, see Supplementary Table S1.

### Data preprocessing

The entire dataset, including sleep diaries and NAPPA data, was first visually reviewed for technical quality. This review resulted in sleep diary data from 104 infants (424 nights) and NAPPA data from 97 infants (372 nights). Paired datasets containing both sleep diary and NAPPA data were available for 363 nights from 99 infants. We then excluded nights where the start or end times in the diary and NAPPA data differed by more than one hour, resulting in a final dataset of 165 nights from 73 infants. A detailed overview of the sample sizes is illustrated in Fig. 1b.

### Defining and detecting NAPPA arousals (NAs)

#### Preprocessing

NAPPA measurement data was stored as csv matrices in the NAPPA sensors and analysed using custom-built scripts in MATLAB (R2023a)^21^ and R Statistical Software (v4.3.2; R Core Team 2023).^22^ In the present study, we only used the accelerometer-based features saved for every 5-s epoch for activity (continuous value) and body position (discrete values). All analyses began 10 min after the bedtime indicated in the sleep diary or later when there was more than 2 min attenuation in the movement activity. In the present study, we focused on 1) detecting the NAPPA Arousals (NAs), and 2) categorising these based on postural changes (calm vs. restless). The mathematical formulas used are described in Supplementary Table S2.

#### NA validation

For better understanding the clinical correlates of NAs, we compared them to awakenings in the clinical polysomnography (PSG) using an additional dataset that included simultaneous NAPPA and PSG registrations in a sleep laboratory. The systematic assessment of these findings is presented in Supplementary data (Supplementary Fig. S2). Overall, our comparison showed that approximately half of the very brief (30 s–1 min) NAs corresponded to PSG-detected awakenings, with this proportion increasing to 70–80% for longer, multi-minute NAs. These findings suggest that not all NAs may reflect cortical arousals. However, their behavioural aspect (overt movement) is still likely relevant from the parental perspective, as brief movements may disturb parents even if they do not fully wake the infant. Therefore, we included a broader range of NA durations in our analyses to better capture sleep fragmentation as it may be experienced by caregivers. For further details, please refer to Supplementary Section 4 of the Supplementary Material.

#### NA detection

To systematically assess nighttime arousals in infants, we used data-driven thresholds across various time windows ranging from 30 s to 10 min. The threshold for high activity was set to twice the median activity level of each night (*n* = 372 nights; mean threshold 0.09 ± 0.007 m/s), such that any activity exceeding this threshold was considered arousal activity. All measurements and detections were visually reviewed to confirm that automated detections generally aligned with an intuitive, visual recognition of arousals in the raw activity signals. We also systematically varied the threshold to confirm robustness of the selected final parameters. Individual arousal events were then pooled into arousal windows using progressively longer time windows (30 s, 1 min, 2 min, 3 min, 5 min, and 10 min) for systematic assessment. To be classified as an arousal, at least half of the activity within each window had to exceed the threshold, regardless of whether the activity was continuous or intermittent. For example, in a 1-min window, if 30 s or more of the activity exceeded the threshold, it would be marked as a NA with the length of the arousal corresponding to the duration of the window being analysed. Consecutive NA windows were combined and counted as one.

The effect of gestational age (GA) on arousal thresholds was studied using GA both as a continuous variable (linear mixed-effects models; *N* = 91 infants; 347 nights) and as categories (non-parametric Kruskal–Wallis test; preterm (*n* = 9), early term (*n* = 15), full term (*n* = 48), late/post-term (*n* = 19)). No significant effects were found with either approach.

#### NA types

We reasoned that arousals in infants may vary in intensity and duration and be detectable with the NAPPA wearable. To explore this, we carried out additional analyses in which all NAs were divided into calm (cNA) and restless (rNA) subtypes based on the frequency of body position changes per minute during the NA window. Specifically, restless arousals (rNA) were defined as those with a frequency of position changes above the third quartile (75th percentile) calculated across all recorded nights (*n* = 372) in our dataset. Calm arousals (cNA), on the other hand, were defined as those that fell below this threshold.

### Statistical analyses

We used several complementary statistical approaches. Median absolute deviation (MAD) was used to quantify arousal variability both within and between individuals. To assess associations between continuous variables, we used Spearman’s rank correlation coefficient, which is robust to non-normal distributions and captures monotonic relationships. To statistically compare the correlation coefficients (strength of associations between pSQ and NAs across sleep settings), we used the *cocor* package. This allowed us to test whether the correlation between pSQ and NAs differed significantly between the two conditions.

Correlation analyses were complemented with Generalised Linear Mixed Models (GLMMs) that can account for repeated measures (multiple nights per infant) and unbalanced datasets (differing numbers of nights per infant, or in some cases, nights with varying sleep settings per infant). To this end, we used participant IDs as a random intercept to control for individual variability, employed a generalised Poisson distribution, used a log link for the conditional mean (μ), and estimated the dispersion parameter (ϕ) from the data. While some individual variability exists, the GLMM results were largely consistent with the Spearman correlations. Thus, we only report GLMMs for the correlation analyses in the main text when they provide additional or meaningfully different insights. Full GLMM results are available in the Supplementary Materials (Supplementary Tables S3–S6) for transparency and completeness. In addition to the correlation-based models, we used GLMM to test whether pSQ and arousals (NAs and RAs) significantly differed by sleep setting. Two GLMM models were used to assess how well pSQ could be explained by arousals, using either RAs or NAs as predictors. For both, Model 1 included arousals only, with random intercepts for participant ID to account for individual variability. Model 2 extended this by adding sleep setting as a fixed effect to assess whether it influenced the relationship. Likelihood ratio tests were used to compare the models and determine whether including the sleep setting significantly improved model fit.

## Results

### Reported arousals and perceived sleep quality: parents’ perspective

**Fig. 2:**
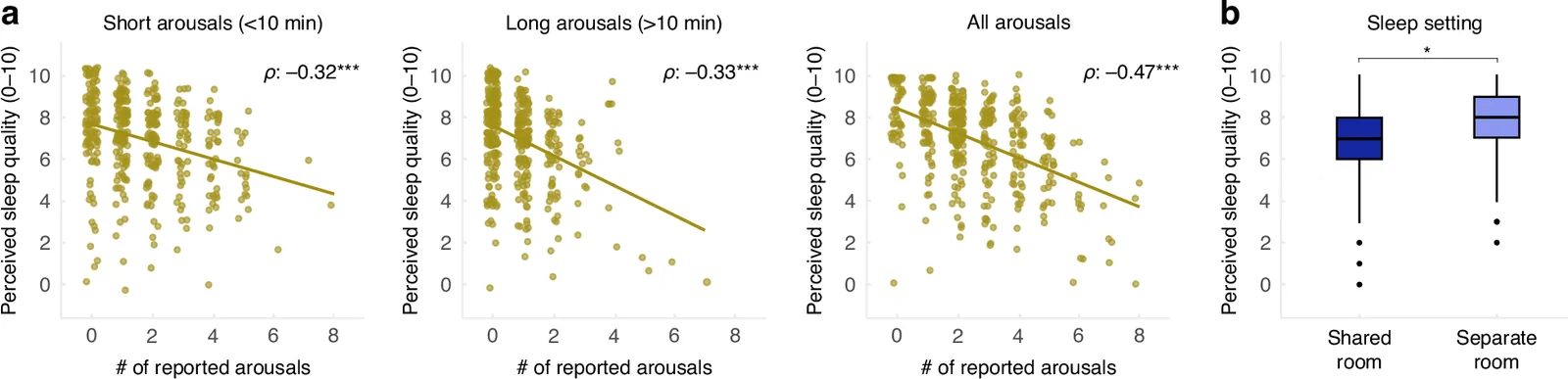
Perceived sleep quality (pSQ) in relation to reported arousals (RAs) and sleep setting. **a** Dot plots showing the correlation between pSQ and short arousals (less than 10 min), long arousals (more than 10 min), and all arousals across individual nights. **b** Comparison of pSQ between nights when infants shared a room with their parents versus slept in a separate room. *p < 0.05, ***p < 0.001.

Sleep diaries from 104 infants showed a significant link between pSQ and the number of reported arousals (RAs; Fig. 2a). Higher numbers of short (ρ = −0.32, *n* = 424, *p* < 0.01), long (ρ = −0.33, *n* = 424, *p* < 0.01), and total arousals (ρ = −0.47, *n* = 424, *p* < 0.01) were each significantly associated with poorer pSQ. In an additional analysis with 43 infants for whom sleep setting was available, pSQ was significantly influenced by sleep setting (Fig. 2b), as indicated by the GLMM analysis (z = −0.12, *n* = 187, *p* = 0.03). Parents reported higher pSQ for infants who slept in a separate room (*n* = 86, mean pSQ = 7.73, SD = 1.91) compared to those who shared a room with their parents (mean *n* = 101, pSQ = 6.80, SD = 1.99).

### NAPPA arousals vs. reported arousals

**Fig. 3:**
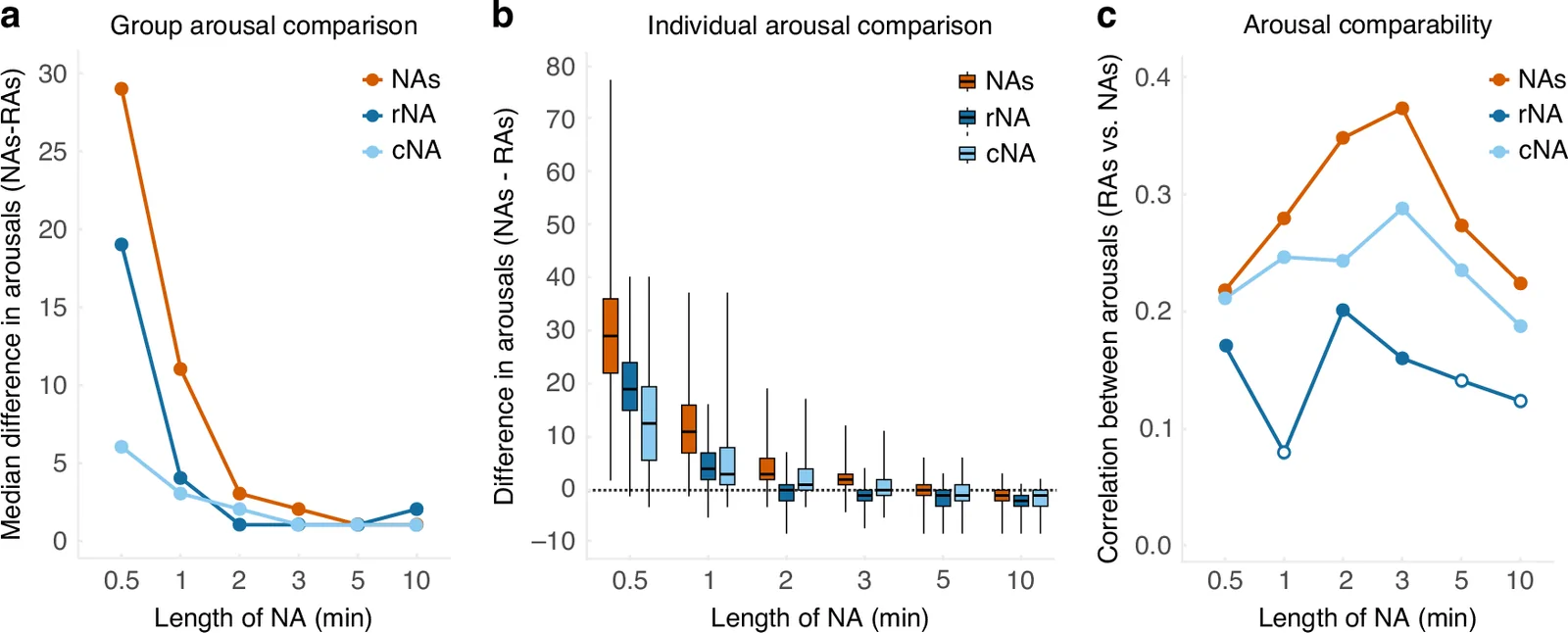
Reported arousals (RAs) vs. NAPPA arousals (NAs). **a** Group-level difference (median absolute deviation) between RAs and NAs across NA lengths and types. **b** Individual-level difference in the number of arousals between RAs and NAs across different NA lengths and types. **c** Correlations between the number of RAs and NAs for different NA lengths and types, with statistically significant correlations shown by solid dots. NAs all NAs, rNA restless NAs, cNA calm NAs.

At the group level (97 infants), the number of short arousals were substantially different between NAs and RAs when using short arousal durations (30 s and 1 min). However, these differences were clearly smaller for longer arousals (2–10 min), as shown in Fig. 3a, which displays the MAD plot. In contrast, at the individual arousal level (Fig. 3b), where NA and RA counts were compared within each recorded night (i.e., per-night comparison), short NAs (30 s or 1 min) were significantly more frequent, while 10-min NAs were consistently fewer than those reported by parents, as illustrated in the boxplots. When examining different NA types, there was a significant correlation between NAs and RAs (Fig. 3c), with the strongest correlation observed for 3-min NA detections (ρ = 0.40, *n* = 165, *p* < 0.001). The correlation weakened for both shorter and longer arousal durations. When we accounted for individual differences, the correlation for the 3-min window was slightly adjusted. However, this change was minimal, suggesting that individual variability has only a small effect on the relationship between NAs and RAs. Despite this adjustment, the 3-min window still showed the strongest correlation, indicating that the general pattern holds even when individual differences are considered. Overall, these findings highlight the challenges parents face in accurately estimating the number of arousals, often underestimating short arousals and/or overestimating longer arousals.

### Effects of sleep setting on NAs and RAs

**Fig. 4:**
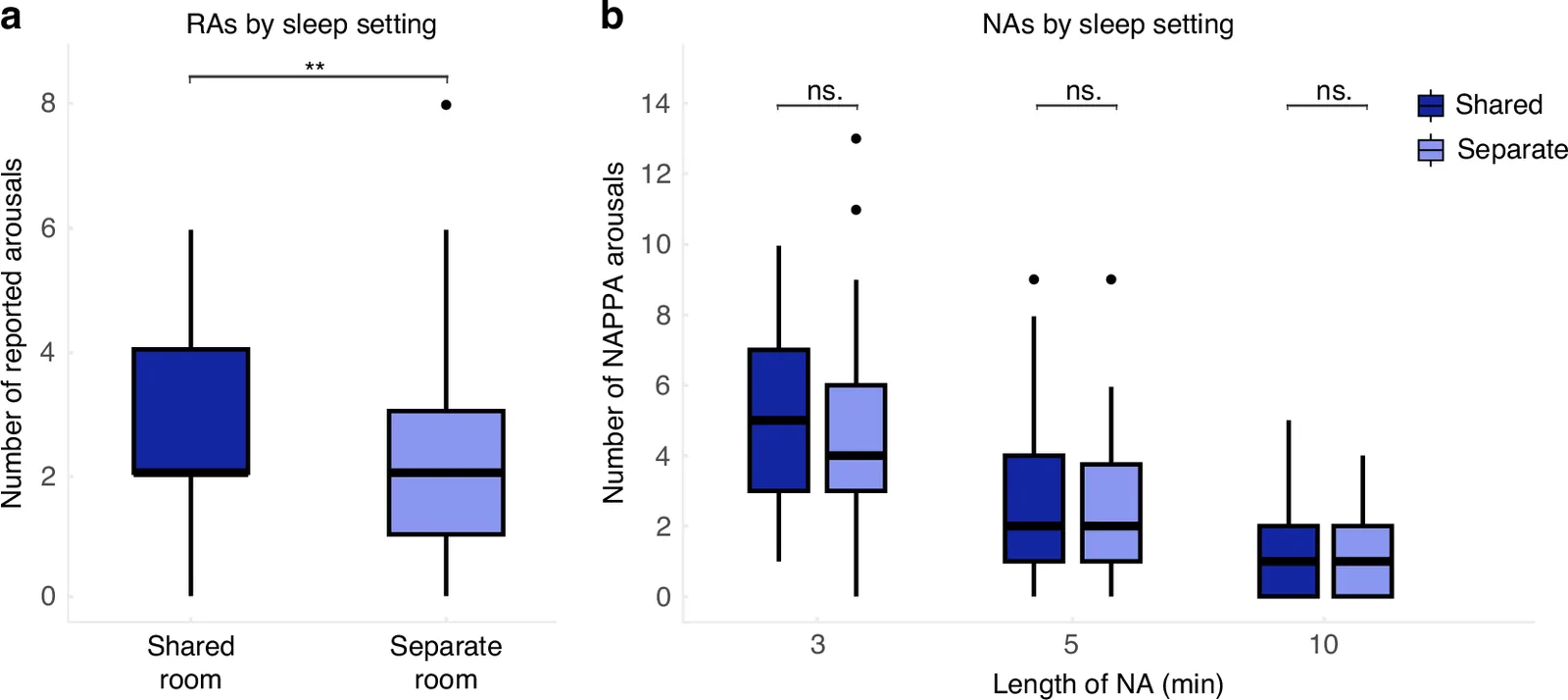
Reported and detected arousals by sleep setting. **a** Parent-reported arousals (RAs) across different sleep settings. **b** NAPPA-detected arousals (NAs) across sleep settings and NA lengths. **p < 0.01, ns non-significant.

**Fig. 5:**
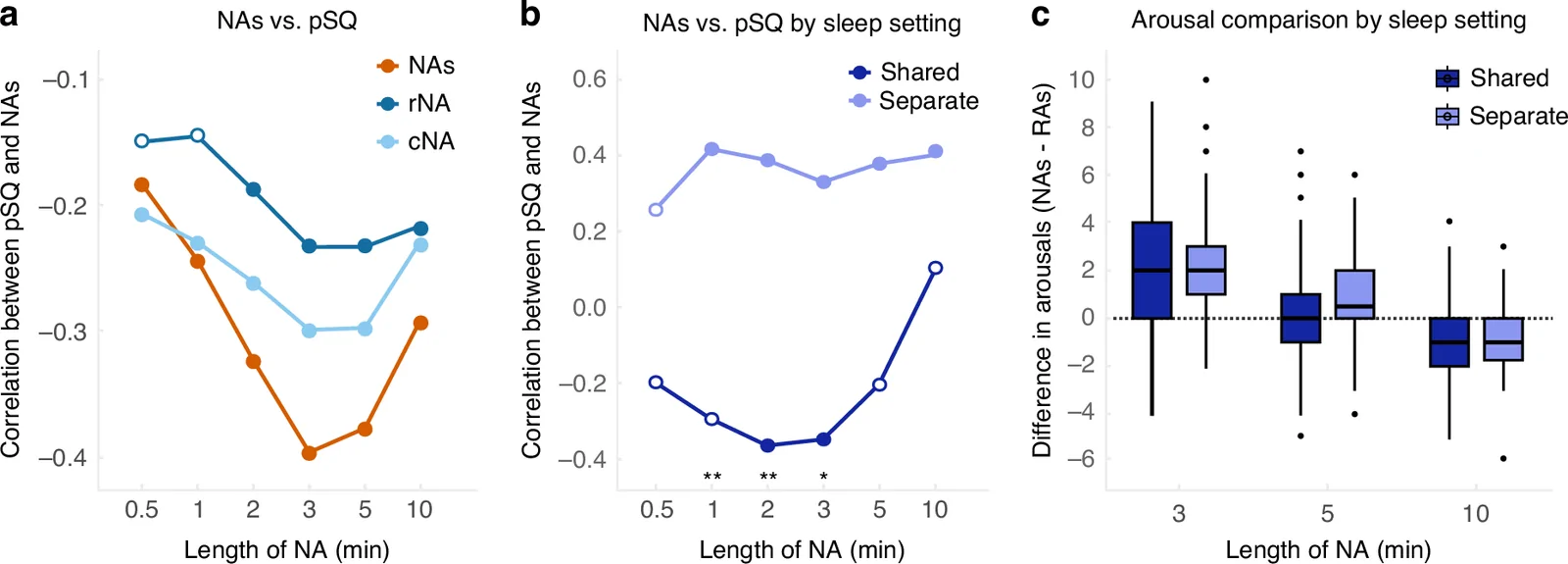
NAPPA arousals (NAs) versus perceived sleep quality (pSQ) and number of nighttime arousals. **a** Correlations between perceived sleep quality (pSQ) and NAs across NA lengths; solid dots indicate significant correlations, and colours represent different NA categories (Orange: all NAs; dark blue: restless NAs; light blue: calm NAs). **b** Correlations between pSQ and NAs by sleep setting and arousal length; asterisks mark significant difference between shared and separate sleep settings (cocor). **c** Differences between reported arousals (RA) and NAs by sleep setting and NA length. *p < 0.05, **p < 0.01

The parentally reported arousals (RAs) were significantly influenced by sleep setting (43 infants), with individual variability considered using GLMM analyses (Fig. 4a; *z* = 2.37, *n* = 187, *p* = 0.02). Parents reported a significantly higher number of arousals when the infant shared the same room with them (*n* = 101, mean RAs = 2.50, SD = 1.47) compared to infants sleeping in a separate room (*n* = 86, mean RAs = 1.91, SD = 1.56). In contrast, NAs (shared room, *n* = 88; separate room, *n* = 70) were not significantly influenced by sleep setting (Fig. 4b). Moreover, a comparison between RAs and NAs of different length (*n* = 158; Fig. 5c) revealed that parents tended to underestimate shorter arousals (3- and 5-min) while overestimating longer arousals in both sleep settings. These observations suggest a significant bias in parental reports of nighttime arousals.

### Impact of sleep setting on pSQ and arousal correlations

A significant correlation was observed between RAs and pSQ (Fig. 2a), but there was also a difference in RAs between sleep settings (Fig. 4a). To provide an objectively measured counterpart to this finding, we first showed that the NAs were significantly correlated with lower pSQ (Fig. 5a) across all arousal durations, most clearly for the 3- and 5-min arousals (ρ = −0.40 to ρ = −0.39; *n* = 165, *p* < 0.001 for both). When accounting for individual differences in the data using GLMM, the association remained, with negative estimates for all NA lengths. Notably, the effect appeared strongest for longer arousal durations, with the estimate shifting from −0.036 (*p* < 0.001) at 3 min to −0.046 (*p* < 0.001) at 5 min and −0.064 (*p* < 0.001) at 10 min. While this pattern slightly differs from the correlation results, where the effect was most pronounced at 3 and 5 min, the overall trend remains the same: a higher number of longer NAs is associated with lower pSQ. This suggests that, while individual variability influences the relationship to some extent, the link between NAs and perceived sleep quality remains robust. More detailed information can be found in Supplementary Table S5.

When examining the relationship between NAs and pSQ based on sleep setting (43 infants, Fig. 5b), an increase in NAs was consistently correlated with lower pSQ in the shared room setting (*n* = 88), with the strongest correlations observed for 3-min (ρ = −0.36) and 5-min (ρ = −0.35) arousals. No comparable association between increased nighttime arousals (NAs) and lower pSQ was observed among infants who slept in a separate room (*n* = 70). While the direct bivariate correlation analyses indicated no statistically significant relationships, our further analysis using GLMM, which accounts for repeated measures and individual variability, suggested consistent, though non-significant negative trends between NAs and pSQ across the longer NA windows in the separate room setting (see Supplementary Table S6). Although the lack of statistically significant relationships between NAs and pSQ remains inconclusive, such an association would be intuitively plausible and suggests the need for larger prospective studies in this area. The pSQ-NAs correlations (*cocor*, Fig. 5b) differed significantly between sleep settings (*n* = 158, *p* < 0.05) for 1- to 3-min arousals. More detailed results are presented in Supplementary Table S7. The GLMM results showed that, for these NA lengths in the shared room setting, an increase in awakenings was significantly (*p* < 0.05) associated with a stronger negative impact on pSQ compared to the separate room setting. This suggests that pSQ is more sensitive to the number of awakenings in the shared room setting than in the separate sleep setting. The night-level comparison (Fig. 5c) further supports this, indicating that parents report arousals more easily when the infant shares the room with them. This aligns with the finding that the number of infants’ measured arousals (NAs) was linked to worse reported pSQ only in the shared-room settings. See Supplementary Material S8 for a detailed breakdown of the results.

Lastly, we examined whether including the sleep setting better explained parents’ subjective views of their infant’s sleep quality (pSQ). The results of GLMMs showed that RAs alone did not significantly explain pSQ (Model 1). Adding information about the infant’s sleep setting (Model 2) resulted in only a slight improvement in explaining pSQ, with a *p*-value for the interaction term of 0.142 (*n* = 187), indicating no significant improvement. In contrast, when examining the three lengths of NAs (1-min, 3-min, and 5-min), we observed a significant improvement after adding sleep setting, as indicated by the p-value of *p* < 0.005 (*n* = 158) for the interaction term. This suggests that sleep setting plays a significant role in explaining pSQ, with stronger associations between NAs and pSQ in the shared room setting compared to the separate sleep setting. Notably, this effect was significant only for objective measurements (NAPPA), not for subjective reports (RAs), suggesting that sleep setting plays a more prominent role when arousals are measured objectively. For more details, see Table 1.

**Table 1:** Summary of GLMM performance for reported arousals (RAs) and NAPPA arousals (NAs) in explaining perceived sleep quality (pSQ).

Model	AIC	BIC	Log-likelihood	Deviance	*p*-value for interaction
RAs					
Model 1	747.14	760.06	−369.57	739.14	
Model 2	747.23	766.62	−367.61	735.23	0.142
NA – 3 min					
Model 1	657.05	669.30	−324.53	649.05	
Model 2	653.45	671.83	−320.73	641.45	0.022*
NA – 5 min					
Model 1	657.05	669.30	−324.53	649.05	
Model 2	654.19	672.57	−321.10	642.19	0.032*
NA – 10 min					
Model 1	663.98	676.23	−327.99	655.98	
Model 2	661.66	680.04	−324.83	649.66	0.042*

Model 1 = Arousals + (1|Participant ID). Model 2 = Arousals + Sleep Setting + (1|Participant ID).

Statistical significance is indicated as *p < 0.05.

## Discussion

Our findings indicate that while parental perception of sleep quality (pSQ) correlates with nighttime arousals, parents’ assessments do not fully align with the infants’ observed arousal patterns measured with NAPPA. Parents tend to underestimate shorter arousals and overestimate longer ones, with these biases varying across different sleep settings. These results are consistent with previous research, which shows that both the total number of arousals^5,23^ and the sleep setting^23,24^ significantly influence parents’ assessments of their infants’ sleep quality. Additionally, our findings align with prior studies indicating that parents report more nighttime awakenings during room-sharing than during solitary sleeping.^18,25,26^ Our study builds on this knowledge by systematically characterising the relationship between infant arousal patterns and parental reports, which appear to provide a selective representation of nighttime events.

This study contributes to the growing body of accelerometer-based research on healthy infants, highlighting the poor agreement between actigraphy and parental reports of awakenings. Some studies suggest that actigraphy may overestimate nighttime arousals compared to PSG,^15,27^ while others indicate that parental reports based on sleep diaries often underestimate awakenings compared to actigraphy.^28,29^ This discrepancy illustrates a complex relationship where both methods have their limitations in measuring sleep disturbances. Much of the inconsistency in prior studies may stem from variability in algorithms and arousal definitions,^30–32^ which are not always described clearly enough to enable direct comparison across the studies. For example, fixed detection thresholds cannot account for differences arising from varying sensor placements, and differing arousal duration criteria may lead to substantially different results. In this study, we addressed these issues by using night-specific, data-driven thresholds, assuming only that the wearable is used consistently on each given night. Additionally, we systematically scanned a wide range of arousal durations, from 30 seconds to 10 minutes, to identify the arousal window that best align with parental reports. Future studies are needed to refine NA detection criteria that align more closely with arousals detected through the clinical gold standard, polysomnography. Nevertheless, both previous actigraphy studies and our current NAPPA measurements consistently reveal a mismatch between parental diaries and objective infant measurements.

Our findings support the common knowledge that infants’ sleep disruptions, arousals or awakenings, may affect parents’ perception of the infants’ sleep quality (pSQ).^6,7^ It is commonly known that parents’ perception of sleep quality (pSQ) reflects night wakings. Our findings support this assumption, as parents reported lower pSQ with a higher number of arousals. This led us to explore the possibility of using direct measurements from the infant to explain parentally perceived poor infant sleep quality. Indeed, wearable-measured NAPPA arousals (NAs) did partially explain pSQ, with a stronger effect when considering the sleep setting. However, even together, these factors explained only a limited portion of pSQ, possibly due to unmeasured variables such as infant crying or parents’ own sleep quality, which we were unable to assess. An intriguing effect of the sleep setting was also observed: parents reported more arousals when sharing the room with their infant, despite no actual difference in the infants’ arousals between sleep settings. While this finding encourages further prospective studies on sleeping arrangements, it is intriguing to note prior literature^33^ has reported better parental sleep rating when infants sleep alone. Altogether, these findings suggest that 1) parentally reported infant sleep quality can only partially reflect the infant’s behaviour as measured objectively, and 2) parental reports may be an unreliable source of information for assessing infant sleep.

The present work has some limitations. Firstly, we measured only nighttime sleep, which is clinically important but may limit the accuracy of diary information. Secondly, there were several nights when we could not obtain fully matching time windows from the diary and NAPPA data. This was due to the sensor’s limited memory capacity, which restricted measurements to fixed 12-h windows that did not always align with families’ sleeping times. Current and future studies will use sensors with larger memory, enabling continuous 24/7 data collection over a week. Thirdly, the retrospective collection of sleep setting information did not cover the full cohort and may be subject to recall bias due to the time elapsed between the original sleep measurement and the follow-up. While the first two limitations may affect the overall accuracy of sleep measurements across the study, the third limitation specifically impacts the robustness of our findings by attenuating the observed group differences between sleep settings. Finally, our study was conducted on infants without reported sleep concerns and within a geographically restricted area, which may limit the generalisability of our findings. However, we would expect that a broader range of sleep concerns (and pSQ) would likely yield effects stronger than those reported in the present study.

The user-friendly and cost-effective design of the NAPPA wearable makes it adaptable for various research and health applications. For instance, it could provide feedback to new parents, help establish healthy sleep routines for children and enhance family well-being. The NAPPA solution can also support broader infant sleep research, including cross-cultural comparisons and exploring the effects of cultural variations on infant sleep. Future studies should combine the present findings with more detailed sleep diaries or analyses of sleep architecture,^19^ which together may provide a clearer view of the factors that shape parental expectations and perceptions of sleep quality. Finally, an at-home wearable like this could easily support therapeutic or academic interventions by identifying targets for intervention or benchmarking efficacy.

## Supplementary information


Supplementary information


## Data Availability

Data can be made available on request according to legal constraints in each use case.
