# Supplementary material for: Assessing infant sleep quality and arousals: comparison of wearable data with parent perspectives
Source: Pediatr Res. 2025 Dec 10;100(1):194–200. doi: 10.1038/s41390-025-04649-y (PMC13524829; doi:10.1038/s41390-025-04649-y)
Supplement: Supplementary file 1 [file 41390_2025_4649_MOESM1_ESM.pdf]

SUPPLEMENTARY MATERIAL

1. Example of Sleep Diary Used
2. Summary of Sleep Measurement Participation
3. Mathematical Descriptions of the Criteria for Arousal Detection and Classification
4. Comparison of arousals detected with NAPPA (NAs) vs Polysomnography (PSG)
5. Analyses accounting for repeated measurements (GLMM)
6. Relationship Between Perceived Sleep Quality (pSQ) and NAs by Sleep Setting

Example of Sleep Diary Used

* Infant sleep diary
Vauvan unipäiväkirja, PILKE-tutkimus

* This diary gathers details about your child's sleep. Please use the same ID for each night.
Tällä päiväkirjalla kerätään yökohtainen tieto lapsesi nukkumisesta. Täytä jokaisen yön osalta tiedot samalla PILKE-projektin antamalla tunnuksella.

*Pakollinen * Obligatory

* ID
Tunnuksesi*: ___________

* Date
Pvämäärä*: ____.____.20___

* Who are you?
Kuka olet?*:

* Caregiver
Huoltaja ○

* Other
Muu ○

* Bedtime
Nukkumaanmenoaika* _____:_____

* Nighttime events (check the box for the most frequent occurrence for each hour)
YÖN tapahtumat* (mitä on ENITEN ko. tuntina? rasti ruutuun)

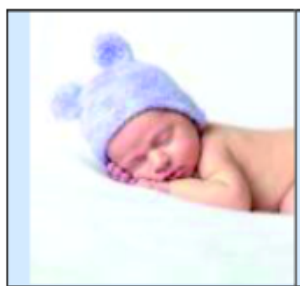	Ei lainkaan	Klo 19-20	20-21	21-22	22-23	23-24	00-01	01-02	02-03	03-04	04-05	05-06	06-07	07-08	08-09
Yrittää unta * Trying to sleep															
Nukkuu * Sleeping															
Havahtuu * Brief awakening hetken< 10min															
Havahtuu * Fully awake valveille>10min															

* Wake-up time
Heräämisaika* _____:_____

* Amount of nighttime wakefulness (estimate)
Yövalvomisen määrä* (sinun arviosi)

ei lainkaan ○ | alle 30min ○ | 30-60 min ○ | 1-2h ○ | yli 2h ○

* Your personal rating of the infant's quality of sleep
Yöunen arvosana omasta mielestäsi*

* Very poor
Erittäin huono 0 ○ 1 ○ 2 ○ 3 ○ 4 ○ 5 ○ 6 ○ 7 ○ 8 ○ 9 ○ 10 ○ Erittäin hyvä
* Very good

* Was there any reason for poor sleep?
Oliko joku syy heikolle unelle?

____

____

* Previous day's naps
Edellisen päivän päiväunet:

1.päiväunijakso (kirjoita unen alku- ja loppuaika. Jos ei päiväunia, mene viimeiseen kysymykseen.)____________________
2.päiväunijakso (kirjoita unen alku- ja loppuaika) ____
3.päiväunijakso (alku- ja loppuaika)____
4.päiväunijakso (alku- ja loppuaika)____

* Would you like to share anything else about last night?
Haluatko kertoa jotain muuta kuluneesta yöstä?

____

____

Figure S1. *Example of the sleep diary used in the study*. The diary was originally completed in Finnish; translations are highlighted in yellow.

Summary of Sleep Measurement Participation

Table S1. Number of Infants and Nights Recorded at 7 and 12 Months of Age

SLEEP DIARY			
At 7 mos		At 12 mos	
N = 77		N = 74	
# of nights		# of nights	
Mean (SD)	Range (IQR)	Mean (SD)	Range (IQR)
2.83 (0.50)	1-3 (0)	2.78 (0.48)	1-3 (0)
NAPPA			
At 7 mos		At 12 mos	
N = 72		N = 69	
Mean (SD)	Range (IQR)	Mean (SD)	Range (IQR)
2.68 (0.62)	1-3 (1)	2.58 (0.69)	1-3 (1)

The table shows the number of infants who participated in the sleep measurement at 7 and 12 months of age. For each time point, the mean and standard deviation (SD), as well as the range and interquartile range (IQR), are reported for the number of recorded nights. The IQR represents the middle 50% of the data, indicating the variability in the number of nights recorded.

Mathematical Descriptions of the Criteria for Arousal Detection and Classification

Table S2. Arousal Detection and Classification Criteria

Parameter	Formula	Definitions
Threshold	$2 \times \text{median}_i$	**Individual threshold:** $median_i$<br>(represents the median value of actigraphy data for each measurement night)
NA	$\textbf{Active}_j = \begin{cases} 1 & \text{if} \sum_{k=1}^{W} I(A_{j,k} > T) \geq 0.5 \cdot W \\ 0 & \text{otherwise} \end{cases}$	**Arousal window:** $Active_j$<br>(indicates whether the j th window is considered arousal)<br>**Window index:** j<br>(index of the window being analysed)<br>**Epoch:** k<br>(index of each individual measurement within the j)<br>**Window length:** W<br>(the number epochs in each window)<br>**Activity levels:** $A_{j,k}$<br>(activity level at epoch k within window j)<br>**Threshold:** T<br>(the value above which $A_{j,k}$ is considered significant)<br>**Indicator Function:** $A_{j,k} > T$<br>(indicator function that is 1 if $A_{j,k}$ exceeds T, and 0 otherwise)
rNA	$\textbf{PCM}_j = \frac{S_j}{C_j + 1} \cdot \frac{1}{60}$<br><br>$\textbf{rNA} = \begin{cases} \text{Yes} & \text{if Active}_j \text{ 1 and PCM}_j > \text{IQR3} \\ \text{No} & \text{otherwise} \end{cases}$	**Movement frequency:** PCM_j<br>(indicates the position changes per minute in the j th window)<br>**Active seconds:** Sj<br>(total number of seconds of arousal windows for the j th night)<br>**Position changes:** Cj<br>(total number of position changes for the j th night)<br>**Restless arousal window:** rNA<br>(indicates whether the j th window is considered restless arousals)
cNA	$\textbf{cNA} = \begin{cases} \text{Yes} & \text{if Active}_j \text{ 1 and PCM}_j < \text{IQR3} \\ \text{No} & \text{otherwise} \end{cases}$	**Calm arousal window:** cNA<br>(indicates whether the j th window is considered calm arousals)

Comparison of arousals detected with NAPPA (NAs) vs. Polysomnography (PSG)

We assessed how arousals detected by NAPPA relate to those detected by conventional clinical polysomnography (PSG). Using an independent cohort[1] of 40 infants aged 0 to 24 months, we detected NAs with different duration criteria from 30 seconds to 10 minutes. We then evaluated how often (proportion) these NAs corresponded to wake scores in PSG. Figure 3, which collates data from the full cohort, shows that nearly half of the shortest NAs (30 seconds) were linked to wake in PSG, with the proportion increasing to 70-80% for NAs lasting 3 minutes or longer.

These findings support the intuitive notion that longer NAs generally align with PSG-defined awakenings. However, they also underscore important differences between behavioural and PSG arousals. The concept of “behavioural arousal” is far broader than its clinical PSG counterpart. Arousals during sleep can occur at various physiological levels, including cortical (EEG), autonomic (e.g., ECG), respiratory, peripheral (EMG), and cognitive (e.g., responsiveness). Our study focuses on infants’ sleep at home and the behavioural arousals that may affect sleep quality for both infants and parents. These may involve not only longer waking periods but also brief movements that do not meet PSG criteria for waking yet may still disturb parents. Therefore, behavioural arousals in infants do not directly correspond to EEG-based arousal definitions used in clinical PSG studies.

[1] de Sena S, Häggman M, Ranta J, et al. NAPping PAnts (NAPPA): An open wearable solution for monitoring Infant’s sleeping rhythms, respiration and posture. *Heliyon*. 2024;10(13):e33295. doi:10.1016/j.heliyon.2024.e33295

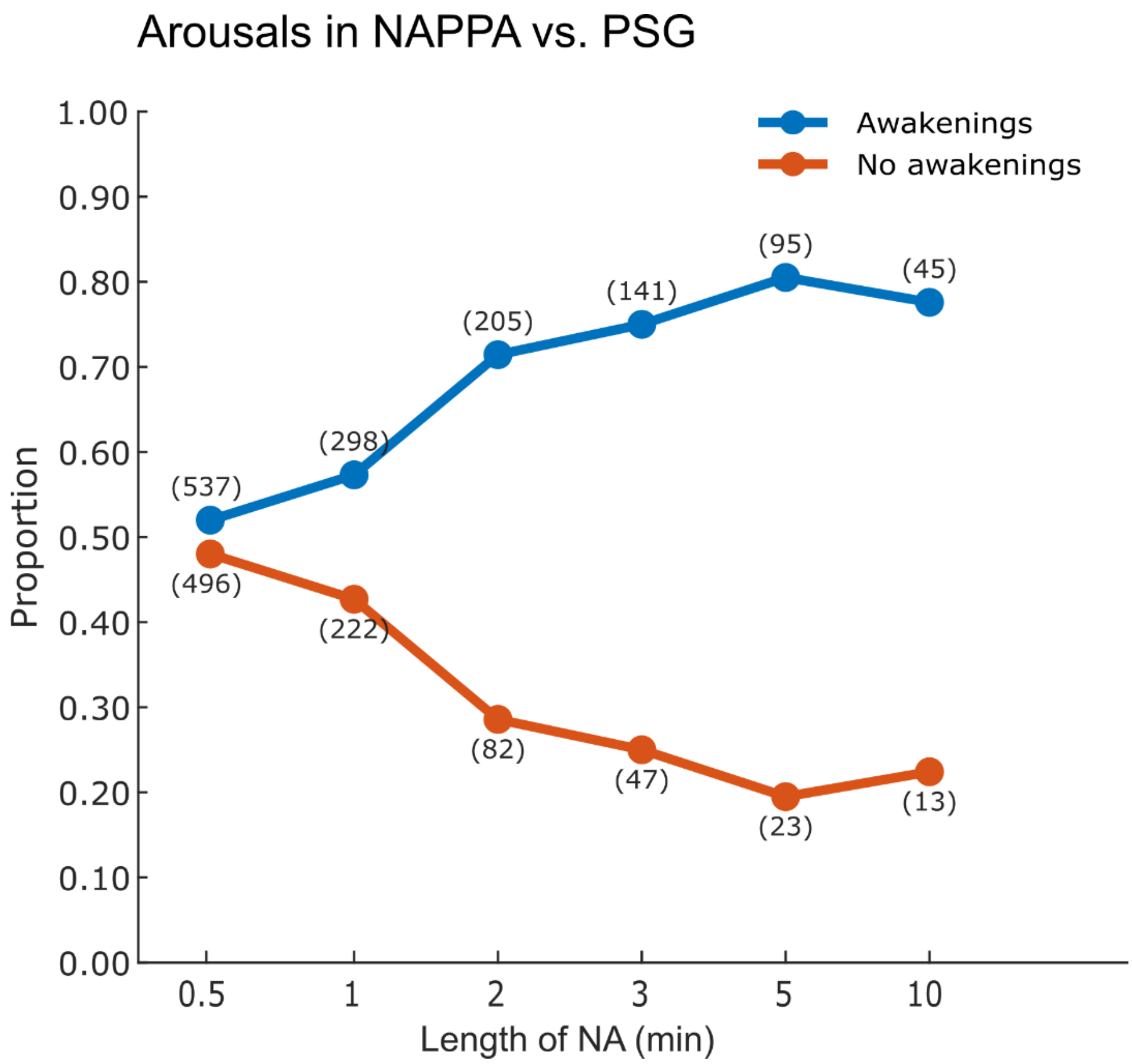

Figure S2. *NAPPA arousals (NAs) vs. polysomnography (PSG) awakenings.* The proportion of NAs corresponding to an awakening (blue) vs. no awakening (red) in the concurrent PSG measurement, with the number of NAs shown in brackets.

Analyses accounting for repeated measurements (GLMMs)

Below are the results from Generalized Linear Models (GLMM) analyses for the correlations presented in Figures 2, 3, and 5. Results that significantly differ between the Spearman correlations (Figures 2, 3, 5) and the GLMM are highlighted in yellow and are further discussed in the main text.

Table S3. Reported Arousals (RAs) vs. perceived Sleep Quality (pSQ)

RA type	Estimate	Standard error	p-value	Variance (Participant ID)	Std. deviation (Participant ID)
**NA**					
Long	-0.100	0.013	<0.001	0.017	0.132
Short	-0.064	0.009	<0.001	0.016	0.127
Total	-0.092	0.008	<0.001	0.018	0.133

Table S4. NAPPA Arousals (NAs) vs. RAs

Length of NA (min)	Estimate	Standard error	p-value	Variance (Participant ID)	Std. deviation (Participant ID)
**NAs**					
0.5	0.014	0.005	0.003	0.255	0.505
1	0.025	0.009	0.003	0.245	0.495
2	0.052	0.015	<0.001	0.231	0.481
3	0.067	0.021	0.001	0.216	0.465
5	0.080	0.028	0.004	0.249	0.499
10	0.134	0.053	0.011	0.258	0.508
**Calm NAs (cNA)**					
0.5	0.018	0.007	0.014	0.245	0.495
1	0.021	0.008	0.012	0.241	0.491
2	0.036	0.016	0.025	0.234	0.484
3	0.042	0.024	0.076	0.229	0.478
5	0.060	0.031	0.049	0.241	0.494
10	0.121	0.062	0.050	0.253	0.503
**Restless NAs (rNA)**					
0.5	0.014	0.008	0.064	0.265	0.515
1	0.008	0.014	0.542	0.263	0.513
2	0.056	0.025	0.025	0.263	0.513
3	0.069	0.030	0.021	0.262	0.512
5	0.087	0.049	0.079	0.262	0.512
10	0.179	0.102	0.094	0.263	0.512

Table S5. NAPPA Arousals (NAs) vs. perceived Sleep Quality (pSQ)

Length of NA (min)	Estimate	Standard error	p-value	Variance (Participant ID)	Std. deviation (Participant ID)
**NAs**					
0.5	-0.004	0.002	0.023	0.012	0.110
1	-0.007	0.003	0.014	0.012	0.110
2	-0.022	0.005	<0.001	0.014	0.118
3	-0.036	0.007	<0.001	0.013	0.113
5	-0.046	0.010	<0.001	0.013	0.112
10	-0.064	0.018	<0.001	0.012	0.108
**Calm NAs (cNA)**					
0.5	-0.004	0.003	0.127	0.012	0.111
1	-0.005	0.003	0.108	0.013	0.112
2	-0.016	0.006	0.006	0.014	0.119
3	-0.024	0.008	0.003	0.013	0.113
5	-0.038	0.011	0.001	0.013	0.115
10	-0.056	0.021	0.008	0.012	0.109
**Restless NAs (rNA)**					
0.5	-0.005	0.003	0.062	0.011	0.105
1	-0.006	0.005	0.201	0.010	0.102
2	-0.024	0.010	0.013	0.010	0.099
3	-0.042	0.012	0.001	0.010	0.101
5	-0.059	0.020	0.002	0.010	0.098
10	-0.113	0.041	0.006	0.010	0.096

Table S6. NAPPA Arousals (NAs) vs. perceived Sleep Quality (pSQ) by Sleep Setting

Length of NA (min)	Estimate	Standard error	p-value	Variance (Participant ID)	Std. deviation (Participant ID)
**Shared**					
0.5	-0.010	0.005	0.058	0.029	0.170
1	-0.014	0.007	0.049	0.256	0.160
2	-0.036	0.013	0.007	0.026	0.160
3	-0.060	0.018	0.001	0.009	0.096
5	-0.058	0.021	0.007	0.012	0.112
10	-0.039	0.044	0.377	0.026	0.160
**Separate**					
0.5	0.002	0.003	0.517	0.007	0.081
1	0.005	0.005	0.257	0.005	0.072
2	0.004	0.011	0.741	0.007	0.083
3	-0.008	0.017	0.646	0.013	0.111
5	-0.016	0.024	0.494	0.014	0.117
10	-0.054	0.043	0.204	0.015	0.121

Relationship Between Perceived Sleep Quality (pSQ) and NAs by Sleep Setting

Table S7. pSQ vs. NA correlations (cfor 1- to 3-minute NA lengths by sleep setting.

Analysis	Statistic	p-value	Change in r (Δr)	95%CI
Separate vs. shared (1 min)	z = 2.74	<0.01	0.63	0.18-1.00
Separate vs. shared (2 min)	z = 2.76	<0.01	0.62	0.18-0.99
Separate vs. shared (3 min)	z = 2.26	<0.05	0.48	0.06-0.86

Table S8. GLMM results for pSQ vs. NA for 1- to 3-minute NA lengths by sleep setting.

Effect	Estimate	Std. Error	z-value	p-value
**1 minute**				
Interaction: NAs x Shared room	-0.019	0.008	-2.225	0.0261
**2 minutes**				
Interaction: NAs x Shared room	-0.038	0.016	-2.293	0.022
**3 minutes**				
Interaction: NAs x Shared room	-0.054	0.024	-2.283	0.022
